# Inter-genus gene expression analysis in livestock fibroblasts using reference gene validation based upon a multi-species primer set

**DOI:** 10.1371/journal.pone.0221170

**Published:** 2019-08-14

**Authors:** Marcelo T. Moura, Roberta L. O. Silva, Pábola S. Nascimento, José C. Ferreira-Silva, Ludymila F. Cantanhêde, Ederson A. Kido, Ana M. Benko-Iseppon, Marcos A. L. Oliveira

**Affiliations:** 1 Departamento de Medicina Veterinária, Universidade Federal Rural de Pernambuco, Pernambuco, Brazil; 2 Departamento de Genética, Universidade Federal de Pernambuco, Pernambuco, Brazil; University of Helsinki, FINLAND

## Abstract

Quantitative reverse transcription PCR (RT-qPCR) remains as an accurate approach for gene expression analysis but requires labor-intensive validation of reference genes using species-specific primers. To ease such demand, the aim was to design and test a multi-species primer set to validate reference genes for inter-genus RT-qPCR gene expression analysis. Primers were designed for ten housekeeping genes using transcript sequences of various livestock species. All ten gene transcripts were detected by RT-PCR in *Bos taurus* (cattle), *Bubalus bubalis* (buffaloes), *Capra hircus* (goats), and *Ovis aries* (sheep) cDNA. Primer efficiency was attained for eight reference genes using *B*. *taurus—O*. *aries* fibroblast cDNA (95.54–98.39%). The RT-qPCR data normalization was carried out for *B*. *taurus* vs. *O*. *aries* relative gene expression using Bestkeeper, GeNorm, Norm-finder, Delta CT method, and RefFinder algorithms. Validation of inter-genus RT-qPCR showed up-regulation of *TLR4* and *ZFX* gene transcripts in *B*. *taurus* fibroblasts, irrespectively of normalization conditions (two, three, or four reference genes). *In silico* search in mammalian transcriptomes showed that the multi-species primer set is expected to amplify transcripts of at least two distinct loci in 114 species, and 79 species would be covered by six or more primers. Hence, a multi-species primer set allows for inter-genus gene expression analysis between *O*. *aries* and *B*. *taurus* fibroblasts and further reveals species-specific gene transcript abundance of key transcription factors.

## Introduction

Cellular states are the result of differential gene expression that arises from stepwise instructive factors during development [1,2]. Alternatively, disease states or phenotypic perturbation of cellular transcriptional programs by environmental or/and genetic factors can be readily identified by gene expression changes [3,4]. Appropriate assessment of transcript abundance is thus paramount for identification of such key cell state regulators and gene expression signatures [3,5].

Quantitative reverse transcription PCR (RT-qPCR) stands out as a method of choice for relative gene expression analysis [6–8]. It allows fast, accurate real-time measurement of gene expression, even for genes with low-transcript abundance [7]. Furthermore, RT-qPCR is also recurrently used to validate gene expression data stemming from other mRNA-based technologies, such as cDNA microarrays and RNA sequencing [6,9,10].

The RT-qPCR data can be presented as an absolute measurement (e.g. copy number per cell) or at a relative expression level basis between two or more experimental conditions [11]. Accurate measurements of relative gene expression by RT-qPCR require internal control genes (i.e., reference genes—RGs) to counterbalance possible bias from technical or experimental factors, such as RNA extraction methods, mRNA integrity, cell-type, developmental stage, ploidy level, and environmental conditions, among others [12–17].Thus adequate choice and validation of RGs remains as one of the most critical steps for the accuracy of the RT-qPCR assay and is mandatory for each experimental design [8,14,18–20].The algorithms built in software packages for RG selection play a major role in the stability value that is calculated for each candidate RG. Moreover, RG stability can be determined by its overall gene expression variation [21], intra-group expression variation [22], based on pair-wise CT analysis [23], by accounting for mRNA integrity [24], or combining multiple algorithms at once [25]. Another substantial progress was made by adopting an RG index rationality [21,26], since no single locus can be efficiently used as an internal control gene across multiple tissues or experimental conditions [27].

With the current relatively ease to generate large-scale gene expression datasets and the increasing demand for high-throughput or automated platforms [6,28,29], the development of tools for various species or technologies can become extremely advantageous. The use of a universal or multi-species primer set for PCR is an attractive approach to shortcut efforts to species-specific primer designing and testing [30–33]. To the best of our knowledge, despite the availability of a report for *Capra hircus* and *Bos taurus* [13], there is no multi-species primer set (MSPS) validated for intra-specific relative gene expression for several livestock species or for inter-genus RT-qPCR analysis. Therefore, the aim was to design a MSPS for selected livestock species that led to validation of RGs in *Ovis aries* and *B*.*taurus* primary cells and for inter-genus relative gene expression via RT-qPCR.

## Material and methods

### Ethics approval

The project was formally approved by the Ethics Commission (CEUA) at the Universidade Federal Rural de Pernambuco (UFRPE) under the license 031/2016, in full accordance with Brazilian law requirements for animal experimentation.

### Primary somatic cell culture

Primary fibroblast cultures were established and propagated as previously described by Moura *et al*. [34]. Ear skin biopsies from artiodactyla males of *B*. *taurus* (cattle), *Bubalus bubalis* (buffaloes), *Capra hircus* (goats), and *O*. *aries* (sheep) were collected at local abattoirs (Pernambuco, Brazil), washed in saline solution (0.9% NaCl) with antibiotics at 35°C, and further washed in 70% ethanol, besides hair removal with a blade. Skin samples were transported to the laboratory in D-PBS containing amphotericin (Fungizone) and antibiotics at 4°C. Skin explants were obtained by slicing each ear biopsy and placing it on 35 or 60 mm Petri dishes. Explants were cultured in high glucose DMEM (Gibco) containing 10% FBS and antibiotics. Fibroblasts were passaged when dishes became confluent and in seven-day intervals afterwards. Early passage fibroblasts (passages 2 and 3) were used for total RNA extraction.

Ovaries of *O*. *aries* were collected at local abattoirs (Pernambuco, Brazil), transported in saline solution (0.9% NaCl) with antibiotics at 35°C, within three hours after slaughter. Cumulus-oocyte complexes (COC) were aspirated from 3–6 mm follicles with an 18G needle into 10 mL syringes, respectively. Follicular content was deposited in Petri dishes in HEPES buffered *in vitro* maturation medium (H-IVM), consisting of TCM-199 with Hank’s salts, 10% fetal bovine serum (FBS), 50 IU mL^-1^ heparin, and 50 μg mL^-1^ gentamicin sulfate. Cumulus cells were isolated from oocytes by gentle pipetting and used for total RNA extraction.

### Total RNA extraction

Primary fibroblasts and cumulus cells (>1.0 x 10^6^) of selected livestock species (*B*. *taurus*, *B*. *bubalis*, *C*. *hircus*, and *O*. *aries*) were washed in 1X PBS (1.15 g Na_2_HPO_4_, 0.2g KH_2_PO_4_, 8.0g NaCl, and 0.2g KCl), snap-frozen in liquid nitrogen, and stored at– 80°C. The total RNA extraction was carried out using Reliaprep RNA Cell Miniprep (Promega), as described by the manufacturer. The total RNA quality (260–280 and 260–230 ratios) and amount was determined by both Nanodrop 2000 C and Qubit (Thermo Scientific), respectively. The RNA samples were evaluated for their integrity by electrophoresis in 1.0% agarose gels, with 70 V, 120 A after a 40-min. run.

### The cDNA synthesis

The reverse transcription (RT) reaction was carried out immediately after total RNA extraction. The procedure was performed with Quantitect RT kit (Qiagen), as described by the manufacturer. Possible residual genomic DNA was removed by the genomic DNA (gDNA) elimination reaction (2 μL gDNA wipeout buffer 7x, 1 μg total RNA and a final volume of 14 μL), incubated at 42°C for three min., and transferred to 4°C immediately. The gDNA elimination reaction was added to the RT reaction (1 μL Quantiscript RT, 4 μL Quantiscript RT buffer 5X, and 1 μL RT primer mix) and incubated at 42°C for 30 min. Moreover, the samples were incubated at 95°C for three min. and stored at—20°C.

### Primer design

Primers were designed for the MSPS using Primer-BLAST [35] and further selected using Primer3plus [36] based on *B*. *taurus*, *C*. *hircus*, or *O*. *aries* EST sequences available in the GenBank (Table 1). Primers were designed for amplicons of 70–200 base pairs (bp), 40–60% GC content, 19–23 bases in size, to contain GC clamps while avoiding primer-dimer formation [36]. Only primers that were predicted to amplify gene transcripts from at least three livestock species (i.e., *B*. *taurus*, *C*. *hircus*, and *O*. *aries*) as assessed by Primer-BLAST were used in the experiment.

**Table 1:** Multi-species primer set to validate reference genes for inter-genus quantitative reverse transcription PCR (RT-qPCR) in *Ovis aries* and *Bos taurus* fibroblasts.

Gene name (symbol)	Function	GenBank Access Number (species)	Primer sequence (F:forward; R:reverse)	Amplicon size (bp)
Actin (ACT)	Constituent of cytoskeleton	JX046106.1 (*Capra hircus*)	F: TGGCACCACACCTTCTACAAC R: GGTCATCTTCTCACGGTTGG	105
ATPase Na+/K+ transporting subunit alpha 1 (ATP1A1)	Ion transport	NM_001009360.1 (*Ovis aries*)	F: GCAGGGGATGAAGAACAAGA R: GAGAAGCGAGTAGGGGAAGG	154
Glyceraldehyde 3-phosphate dehydrogenase (GAPDH)	Glycolysis	gi27525390 (*Capra hircus*)	F: TGGAGGGACTTATGACCACTG R: AGAAGCAGGGATGATGTTCTG	119
H3 histone, family 3A (H3F3A)	DNA binding protein	NM_001014389.2 (*Bos taurus*)	F: ACTGGAGGGGTGAAGAAACC R: CCTCACTTGCCTCCTGCAAA	199
Peptidylprolyl isomerase A (PPIA)	Cyclosporine binding protein	NM_001308578.1 (*Ovis aries*)	F: GACTGAGTGGTTGGATGGCA R: GCCATTTCTGGACCCAAAGC	97
Ribosomal proteinL19 (RPL19)	Protein synthesis	gi94966830 (*Bos taurus*)	F: ATGAAATCGCCAATGCCAAC R: GGCAGTACCCTTTCGCTTACC	167
Succinate d. complex flavoprotein subunit A (SDHA)	Mitochondrial respiratory chain	XM_012125144.1(*Ovis aries*)	F: GGAGCTGGAGAATTACGGCA R: CGCAGGGACCTTCCATACAA	177
TATA-binding protein (TBP)	Regulation of transcription	XM_012166509.1 (*Ovis aries*)	F: AGAATAAGAGAGCCCCGCAC R: TTCTTCACTCTTGGCTCCCG	78
UbiquitinB (UBB)	Protein degradation	NM_001009202.1 (*Ovis aries*)	F: GCATTGTTGGGTTCCTGTGTC R: CACGAAGATTTGCATTTTGAC	98
tyrosine 3- monooxygenase/tryptophan 5-m.activation protein zeta (YWHAZ)	Signal transduction	NM_174814.2 (*Bos taurus*)	F: CCGGACACAGAACATCCAGTC R: CTCCAAGATGACCTACGGGC	200

### Reverse transcription polymerase chain reaction (Rt-PCR)

The RT-PCR reactions for the MSPS using livestock fibroblast cDNA (i.e., *B*. *taurus*, *B*. *bubalis*, *C*. *hircus*, and *O*. *aries*) were carried out in a 96 reaction thermal cycler (Techne), as previously described by Moura *et al*. [34]. The reaction was composed of 1 μL cDNA (non-diluted samples), 0.6 μL of each primer (2.5 μM), 2 μL 10 X PCR Buffer, 0.2 μL dNTP (10 mM), 0.6 μL 50 mM MgCl_2_, 0.1 μL Taq Polymerase (LGC Biotecnologia), and 14.9 μL ultra-pure H_2_O to a final volume of 20 μL. The cDNA was denatured, and Taq polymerase was activated at 94°C for 5 min. Moreover, 35 PCR cycles were carried out with the following conditions: denaturation at 94°C for 1 min., annealing at 58°C (*C*. *hircus* and *O*. *aries*) or 60°C (*B*. *taurus* and *B*. *bubalis*) for 1 min., and extension at 72°C for 1 min. The final extension step was performed at 72°C for 10 minutes. Amplicon visualization was carried out by electrophoresis in 0.5X TBE buffer and 1.5% agarose gels with 70 V, 120 A for 20 min.

### Quantitative reverse transcription PCR (RT-qPCR)

The RT-qPCR reactions were performed in Line Gene 9660 FQD-96A (Bioer), using SYBR Green detection system. The reaction composed of 1 μL cDNA, 5 μL 2x Go Taq qPCR Master Mix (Promega), 0.6 μL primers (2.5 μM), and 3.4 μL ultra-pure H_2_O to a final volume of 10 μL. Both fibroblast and cumulus cell cDNA were used as diluted samples (1:10). The reactions were carried out under the following conditions: initial denaturation at 95°C for 2 min., 40 cycles at 95°C for 15 seconds, and 58°C for 60 seconds (*O*. *aries* cumulus cells cDNA) or 60°C for 60 seconds (*O*. *aries* fibroblast cDNA or *B*. *taurus* fibroblast cDNA) for relative gene expression analysis. Samples of bulk fibroblast cDNA (1:1 *O*. *aries—B*. *taurus*) were initially used for determining primer efficiency in a similar fashion to as previously described for sheep-goat hybrid embryos [37], followed by species-specific primer efficiency testing (using non-mixed cDNA) and relative gene expression analysis (S1 Fig). Melting curves were analyzed from 65 to 95°C for 20 min. after the initial 40 cycles. Expression levels of candidate genes were evaluated by the cycle of quantification (Cq) during the exponential phase (log) of the PCR. Primer amplification efficiency (E = 10^−1^ / slope), correlation coefficient (R^2^), and interception (y) were determined by the standard curve method using cDNA serial dilutions: 10^0^ (non-diluted samples), 10^−1^, 10^−2^, 10^−3^, and 10^−4^ [38]. The Minimum Information for Publication of Quantitative Real-Time PCR Experiments (MIQE) guidelines was followed to increase both the transparency and reliability of the results generated in the study [12,14,19,20].

The relative gene expression was obtained with three biological replicates and three technical replicates. The relative gene expression levels were evaluated with the REST tool (version 2.0.13) that relies on the 2^-ΔΔCT^ method [11,39], which is based on pairwise comparisons using randomization and bootstrapping techniques—Pairwise Fixed Reallocation Randomization Test [40].

### RT-qPCR data normalization

The BestKeeper algorithm uses raw Cq values as input [21], and calculates the standard deviation (SD) from Cq values for each RG. The RGs with >1.0 SD are considered inconsistent and should be removed from the analysis. By a Pearson correlation coefficient (geometric mean of Cq values of candidate genes) the BestKeeper (version 1) identifies the most stable RGs, defining the RG index for RT-qPCR data normalization [21].

The model-based approach to estimation of expression variation by the NormFinder algorithm ranks the most suitable genes from the candidate RG set [22] while considering both intragroup and intergroup variations. Further, the NormFinder (version 0.953) identifies the most stable RG and the two most appropriate RG by pair-wise comparisons. In turn, the GeNorm algorithm (version 3.5) calculates the RG stability measurement (M) as the average pairwise variation of each RG with all the other RGs [26]. It also enables the elimination of less stable RGs and reassessment of M-values, resulting in the final ranking of RGs. The RGs with lower M-value hold more stable expression among samples [26]. Moreover, GeNorm algorithm also calculates the RG index after inclusion of additional RGs to the most stable RG pair initially found (Pairwise variation—*V*).

The delta-CT method was also used for ranking candidate RGs [23]. This algorithm is based on a simple pairwise delta-CT measurement in each sample. The readout of the delta-CT method is a RG ranking from the most stable to the least stable candidate RG. The raw RT-qPCR data was also subject to the RefFinder algorithm to combine the results from the aforementioned software packages [25], which is available online at the following link (https://www.heartcure.com.au/for-researchers/).

### *In silico* analysis of primer specificity and candidate reference gene expression in somatic tissues

RNA sequencing (RNA-Seq) data for each candidate RG of both *B*. *taurus* and *O*. *aries* was retrieved from gene expression atlas at the European Bioinformatics Institute (https://www.ebi.ac.uk/gxa/home). Only RNA-Seq experiments that contained common tissues in both species were analyzed and described in a side-by-side fashion.

*In silico* analysis of primer specificity was carried out using Primer-BLAST [35]. Each primer of the MSPS was subject to BLAST to mammalian transcriptomes in the GenBank database (Tax-id: 40674) or selected non-mammalian species (*Anolis carolinensis*, *Danio rerio*, *Drosophila melanogaster*, *Gallus gallus*, and *Xenopus laevis*). A maximum of two mismatches in each primer was tolerated for positive hits. The Primer-BLAST search was performed in 05.24.2018. All results were curated manually.

## Results

### Designing a multi-species primer set and testing its specificity in selected livestock species by RT-PCR and RT-qPCR

Total RNA was initially retrieved for two-step RT-qPCR (S2 Fig). The experimental design for primer design and validation (S3 Fig) was effective at obtaining primers for all ten candidate gene transcripts (Table 1). Based on *in silico* analysis, all primers expected to lead to specific PCR reactions in *B*. *taurus*, *C*. *hircus*,and *O*. *aries*. The primer design also allowed primers annealing at conserved sequences (S3 Fig), covering multiple deposited sequences of each loci in *B*. *taurus*, *B*. *bubalis*, *C*. *hircus*, and *O*. *aries* (S3 Fig).

**Fig 1:**
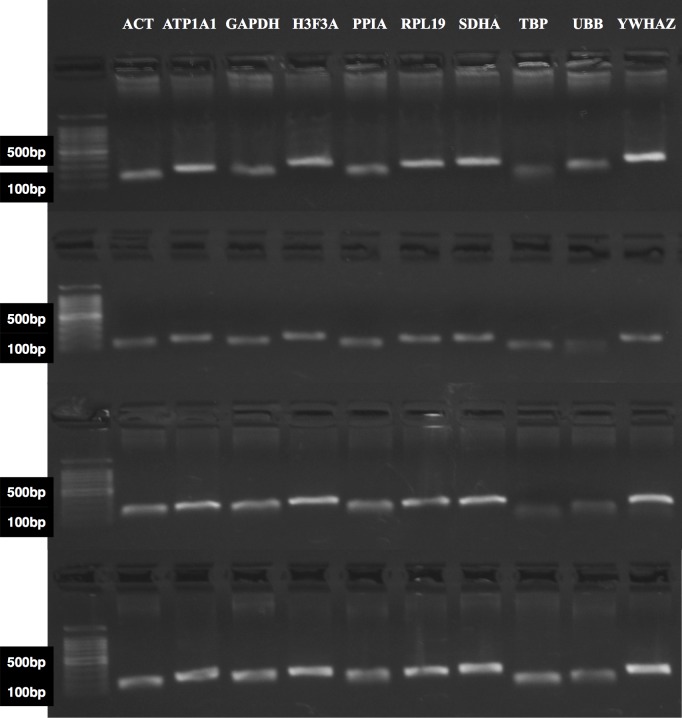
Detection of gene transcripts from the multi-species primer set (MSPS) in *C*. *hircus*, *O*. *aries*, *B*. *taurus*, and *B*. *Bubalis* fibroblast cDNA by reverse transcriptase polymerase chain reaction (RT-PCR). Molecular weight: 100 base pair (bp).

All ten gene transcripts of the MSPS detected in *O*. *aries* cDNA from primary fibroblasts, displaying single amplicons with their expected sizes (Fig 1). Under identical PCR conditions, the MSPS was also detected in *C*. *hircus* fibroblasts samples (Fig 1). Moreover, adjusting annealing temperature allowed the detection of single amplicons for all ten gene transcripts of the MSPS in *B*. *taurus* and *B*. *bubalis* fibroblast cDNA (Fig 1). Additionally, the MSPS was also detected as absolute RT-qPCR reactions (*B*. *taurus*, *B*. *bubalis*, *C*. *hircus*, and *O*. *aries*), as demonstrated by the merged melting curves from qPCR reactions of these four *species*. Of note, subtle differences in melting curves may have arisen from varying isoform amplification or species-specific amplification efficiency (S4 Fig).

### Primer efficiency for RT-qPCR using a multi-species primer set and cDNA from *O*. *aries* and *B*. *taurus* primary somatic cells

Primer efficiency was determined using *B*. *taurus—O*. *aries* fibroblast cDNA (Table 2). From the ten primers, eight were efficient (95.54% - 98.39 while *SDHA* and *TBP* did reach this threshold, and were therefore removed from the data representation (Table 2), being not further investigated. The correlation coefficient varied from—0.990 to—0.999, with slopes from—3.37 to—3.43, and Y intercepts from 26.74 to 31.33 (Table 2). To exclude the potential of bulked cDNA-based bias, primer efficiency was also tested for a subset of primers using non-mixed *O*. *aries* and *B*. *taurus* fibroblast cDNA (S4 Table). Furthermore, primer efficiency in *O*. *aries* varied from 90.69% - 103.07%, while *B*. *taurus* primer efficiency ranged from 92.26–102.04%. The variation for correlation coefficient (- 0.990 to—0.999), slope (- 3.25 to—3.57), and Y intercepts (24.74 to 32.59) were slightly higher using non-mixed cDNA in comparison to the bulk cDNA approach (S4 Table). In turn, the determination of primer efficiency using *O*. *aries* cumulus cDNA found that nine primers were efficient (90.69% - 110.76%), with the exception of *H3F3A* (S1 Table). Also using sheep cumulus cDNA, the correlation coefficient varied from—0.995 to—0.999, with slopes from—3.09 to—3.57, and Y intercepts from 24.74 to 31.83. All primers of the MSPS were efficient in at least one experimental condition or cell type, thus suggesting that their efficiency was dependent on cell type-specific unidentified factors rather than limitations stemming in primer design.

**Table 2:** Primer efficiency, coefficient correlation, slope, and Y intercept derived from the standard curve of each candidate reference gene from a multi-species primer set using *O*. *aries*—*B*. *taurus* fibroblast cDNA via RT-qPCR assay.

Gene	E (%)	NTC (Cq)	Correlation Coefficient (R)	Slope	Y intercept
**Reference genes**					
ACT	97.24	-	- 0.997	-3.39	29.05
ATP1A1	98.04	-	- 0.995	-3.37	31.32
GAPDH	97.55	-	- 0.995	-3.38	31.33
H3F3A	95.62	-	-0.997	-3.43	27.98
PPIA	95.54	-	-0.999	-3.43	28.47
RPL19	96.64	-	-0.990	-3.41	26.74
UBB	97.89	-	-0.999	-3.37	29.34
YWHAZ	98.39	-	-0.994	-3.36	30.17
**Genes of Interest**					
TLR4**	103.65	-	-0.999	-3.24	37.60
TLR4**	109.52	-	-0.987	-3.11	35.85
ZFX	105.77	-	-0.992	-3.19	33.23

**TLR4 efficiency determined separately (*B*. *Taurus* and *O*. *aries* cDNA) since bulk cDNA did not attain acceptable primer efficiency. Actin (ACT), ATPase Na+/K+ transporting subunit alpha 1 (ATP1A1),Glyceraldehyde 3-phosphate dehydrogenase (GAPDH), H3 histone, family 3A (H3F3A), Peptidylprolyl isomerase A (PPIA), Ribosomal protein L19 (RPL19), Succinatedehydrogenasecomplexflavoproteinsubunit A (SDHA), TATA-binding protein (TBP), Ubiquitin B (UBB), Tyrosine 3—monooxygenase / tryptophan 5—monooxygenase activation protein zeta (YWHAZ). Cq: Cycle of quantification. E: efficiency. N.T.C.: No template control.

### RT-qPCR data normalization

The BestKeeper algorithm ranked the seven RG candidates by their Pearson coefficient (Table 3). However, both *RPL19* and *YWHAZ* were considered inconsistent due to their high standard deviation (1.18 and 1.16) and removed from the analysis (Table 3). Based on the Pearson coefficient, ATP1A1 and UBB showed higher values than *GAPDH*, *PPIA*, and *ACT*, thus leading to a more stable RG index as a pair.

**Table 3:** Standard deviations (SD) and Pearson’s correlations obtained from RT-qPCR data using the BestKeeper algorithm.

Gene	ACT	ATP1A1	GAPDH	PPIA	RPL19	UBB	YWHAZ
**Ranking**	7	1	5	6	3	4	2
**Standard Deviation**	0.80	0.93	0.93	0.50	**1.18**	0.67	**1.16**
**Pearson Coefficient**	0.517	0.968	0.819	0.806	0.934	0.912	0.939
**P Value**	0.085	0.001	0.001	0.001	0.001	0.001	0.001

Actin (ACT), ATPase Na+/K+ transporting subunit alpha 1 (ATP1A1), Glyceraldehyde 3-phosphate-dehydrogenase (GAPDH), H3 histone, family 3A (H3F3A), Peptidylprolyl isomerase A (PPIA), Ribosomal protein L19 (RPL19), Succinate dehydrogenase complex flavoprotein subunit A (SDHA), TATA-binding protein (TBP), Ubiquitin B (UBB), Tyrosine 3-monooxygenase/tryptophan 5-monooxygenase activation protein zeta (YWHAZ).

The RT-qPCR data normalization using NormFinder algorithm found ATP1A1 as the most stable RG (0.011). The best RG pair using the NormFinder was the same as the BestKeeper algorithm, namely *ATP1A1* and *UBB* (0.010). Moreover, the RG ranking by NormFinder was substantially different from BestKeeper (Table 4), although three out of the four most stable were shared by both algorithms.

**Table 4:** Stability values derived from RT-qPCR data using *B*. *taurus* and *O*. *aries* fibroblasts determined by NormFinder and GeNorm algorithms.

Gene	NormFinder		GeNorm	
		Ranking		Ranking
ATP1A1	0.011	1	0.734	1
UBB	0.021	2	0.766	2
PPIA	0.022	3	0.865	3
YWHAZ	0.032	4	0.879	4
GAPDH	0.035	5	0.914	6
RPL19	0.045	6	0.894	5
ACT	0.048	7	1.175	7

Actin (ACT), ATPase Na+/K+ transporting subunit alpha 1 (ATP1A1), Glyceraldehyde 3-phosphatedehydrogenase (GAPDH), H3 histone, family 3A (H3F3A), Peptidylprolyl isomerase A (PPIA), Ribosomal protein L19 (RPL19), Succinate dehydrogenase complex flavoprotein subunit A (SDHA), TATA-binding protein (TBP), Ubiquitin B (UBB), Tyrosine 3-monooxygenase/tryptophan 5-monooxygenase activation protein zeta (YWHAZ).

**Fig 2:**
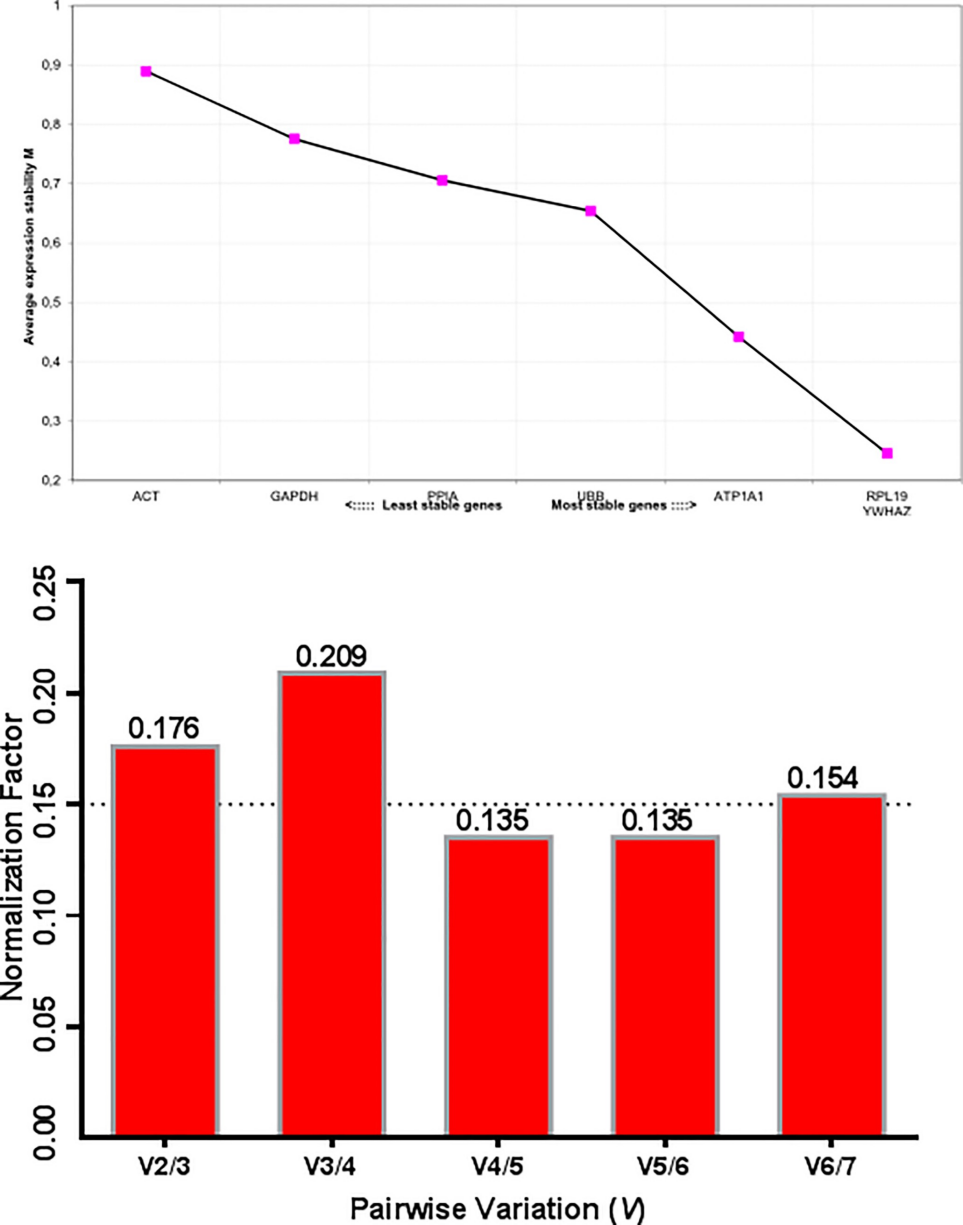
Average gene expression stability values (*M*) of seven potential reference genes from the multi-species primer set (MSPS) for normalization of inter-genus RT-qPCR data based on the GeNorm analysis (A). Pairwise variation—*V* and inter-genus analysis of seven potential reference genes for RT-qPCR data normalization (B).

The GeNorm algorithm ranked the RGs in a similar way as the NormFinder (Table 4). After the GeNorm RG pairwise analysis, *RPL19* and *YWHAZ* was the most stable RG pair (Fig 2). The pairwise variation (*V*) analysis showed that increasing the number of RG to three and four, also increases the RT-qPCR data normalization efficiency (Fig 2).

**Fig 3:**
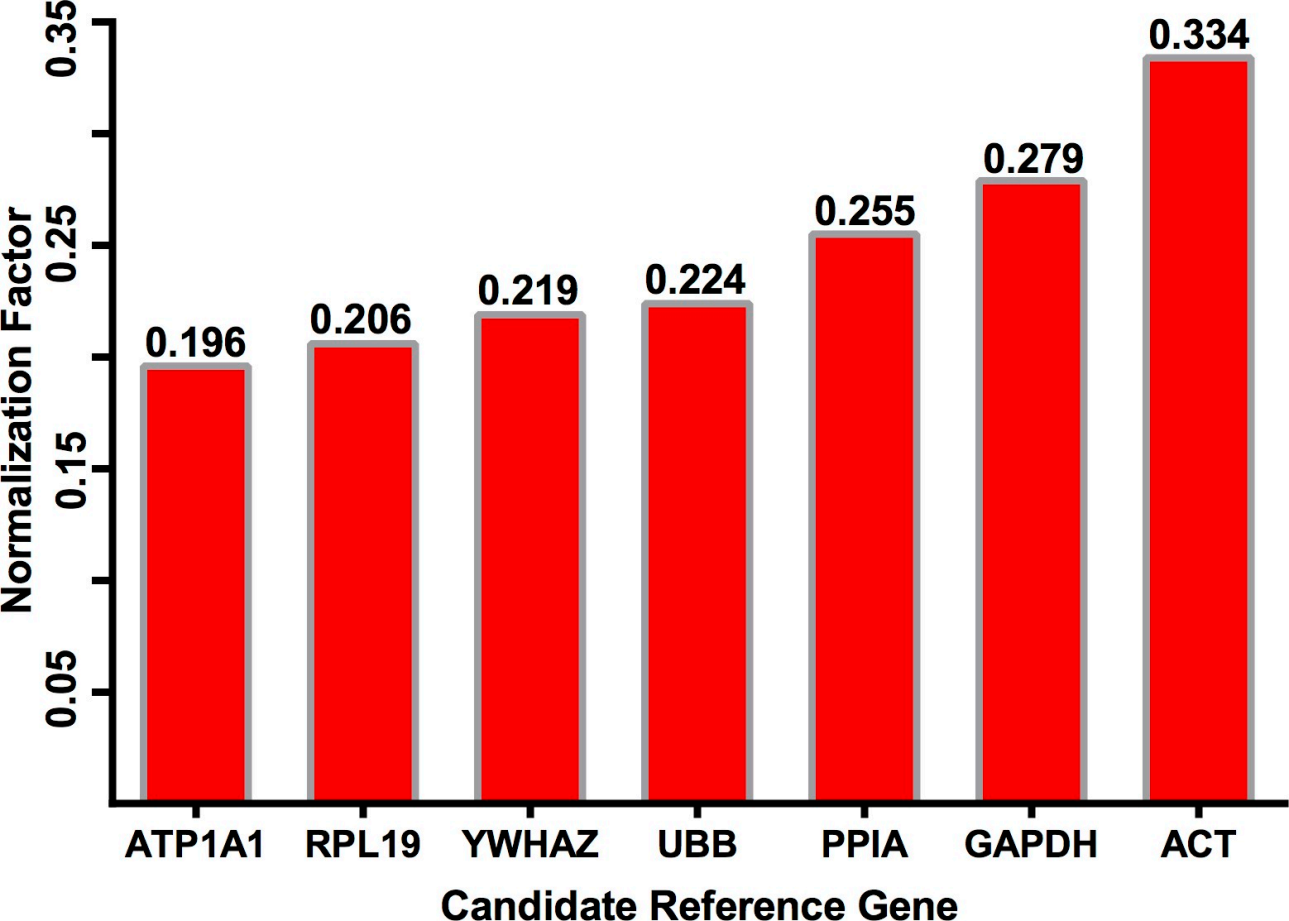
Reference gene ranking by the delta-CT method for normalization of *B*. *taurus*–*O*. *aries* inter-genus RT-qPCR.

The delta-CT method ranked *ATP1A1*, *RPL19*, *YWHAZ*, and *UBB* as the four best ranked RGs (Fig 3). It is noteworthy that RG ranking by the delta-CT method was substantially different from GeNorm and NormFinder algorithms (Fig 3), but more in agreement with BestKeeper. Both *ACT* and *GAPDH* were the two least stable candidate RGs (Fig 3).

**Fig 4:**
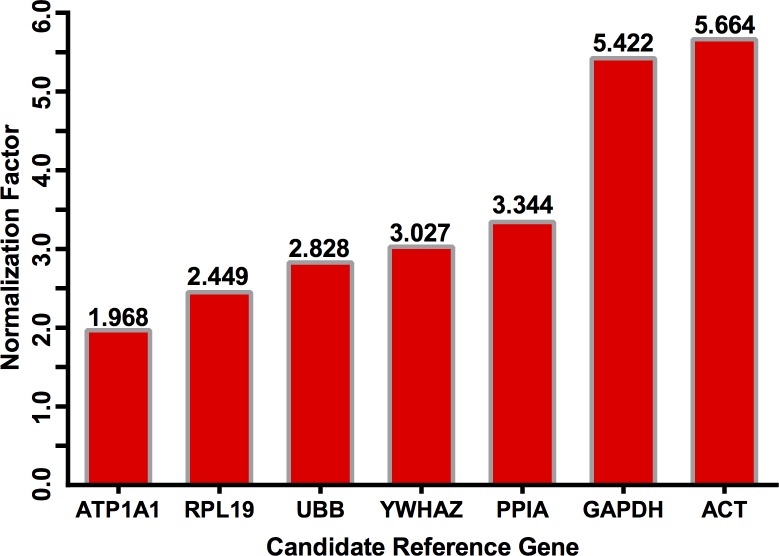
Reference gene ranking by the RefFinder algorithm for normalization of *B*. *taurus*–*O*. *aries* inter-genus RT-qPCR.

The RefFinder algorithm ranked *ATP1A1*, *RPL19*, *UBB*, and *YWHAZ* as the top four most stable RGs (Fig 4), thus very closely overlapping the delta-CT method (Fig 3). The exceptions to this rule are *YWHAZ* and *UBB*, which ranked interchanging at the third and fourth between these two algorithms.

### Validation of inter-genus RT-qPCR using Toll-like receptor 4 (TLR4) and Zinc finger protein, X-linked (ZFX) gene transcripts

**Fig 5:**
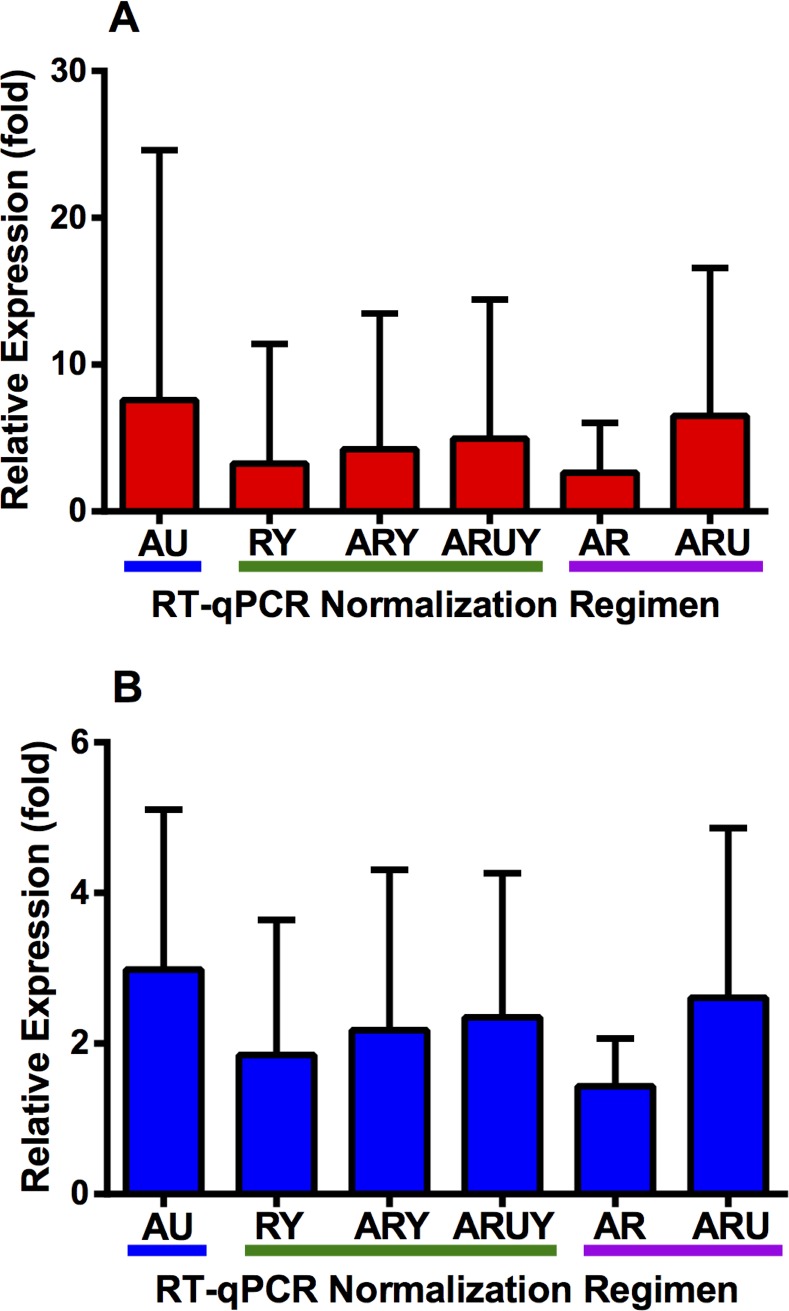
Relative expression of *Toll-like receptor* 4—*TLR4* (A) and *Zinc finger protein*, *X-linked*—*ZFX* (B) gene transcripts determined by the REST software using the 2^-ΔΔCT^ methodology in cDNAs from *O*. *aries* and *B*. *taurus* fibroblasts via inter-genus RT-qPCR analysis. A: ATPase Na+/K+ transporting subunit alpha 1 (ATP1A1). R: Ribosomal protein L19 (RPL19). U: Ubiquitin B (UBB). Y: Tyrosine 3-monooxygenase/tryptophan 5-monooxygenase activation protein zeta (YWHAZ). Blue line: NormFinder and Bestkeeper-based RT-qPCR normalization. Green line: GeNorm RT-qPCR normalization using 2, 3, and 4 genes. Purple line: Other combinations of reference genes for RT-qPCR normalization.

Validation of inter-genus relative gene expression via RT-qPCR was carried out for *TLR4* and *ZFX* using four RGs (*ATP1A1*, *RPL19*, *UBB*, and *YWHAZ*), under varying normalization regimens. Using the two most stable RGs found by BestKeeper and NormFinder algorithms (i.e., *ATP1A1* and *UBB*), *ZFX* was found to be induced in *B*. *taurus* by 2.98 fold, while *TLR4* was also up-regulated by 7.59 fold (Fig 5). The RT-qPCR normalization using the best RG pair found by the GeNorm algorithm (*ATP1A1* and *RPL19*), showed that candidate genes display a milder but consistently higher expression in *B*. *taurus* fibroblasts (*ZFX*: 1.43 fold and *TLR4*: 2.63 fold). The addition of a third and fourth RGs to the best RG pair found by GeNorm increased the relative gene expression difference between species (Fig 5). Collectively, the results suggest that both candidate genes are up-regulated in *B*. *taurus* fibroblasts in comparison to their *O*. *aries* counterparts.

### *In silico* approach for assessing the multi-species primer set for several *B*. *taurus* and *O*. *aries* tissues and for other mammals

To better estimate the potential of the MSPS for normalizing RT-qPCR data in *B*. *taurus* and *O*. *aries*, RNA-Seq data was retrieved for seven organs (tissues) from an available gene expression atlas (S5 Fig). The most stable RGs above (ATP1A1, RPL19, UBB, and YWHAZ) are detected in most tissues, except muscle of *B*. *taurus* (S5 Fig).

**Fig 6:**
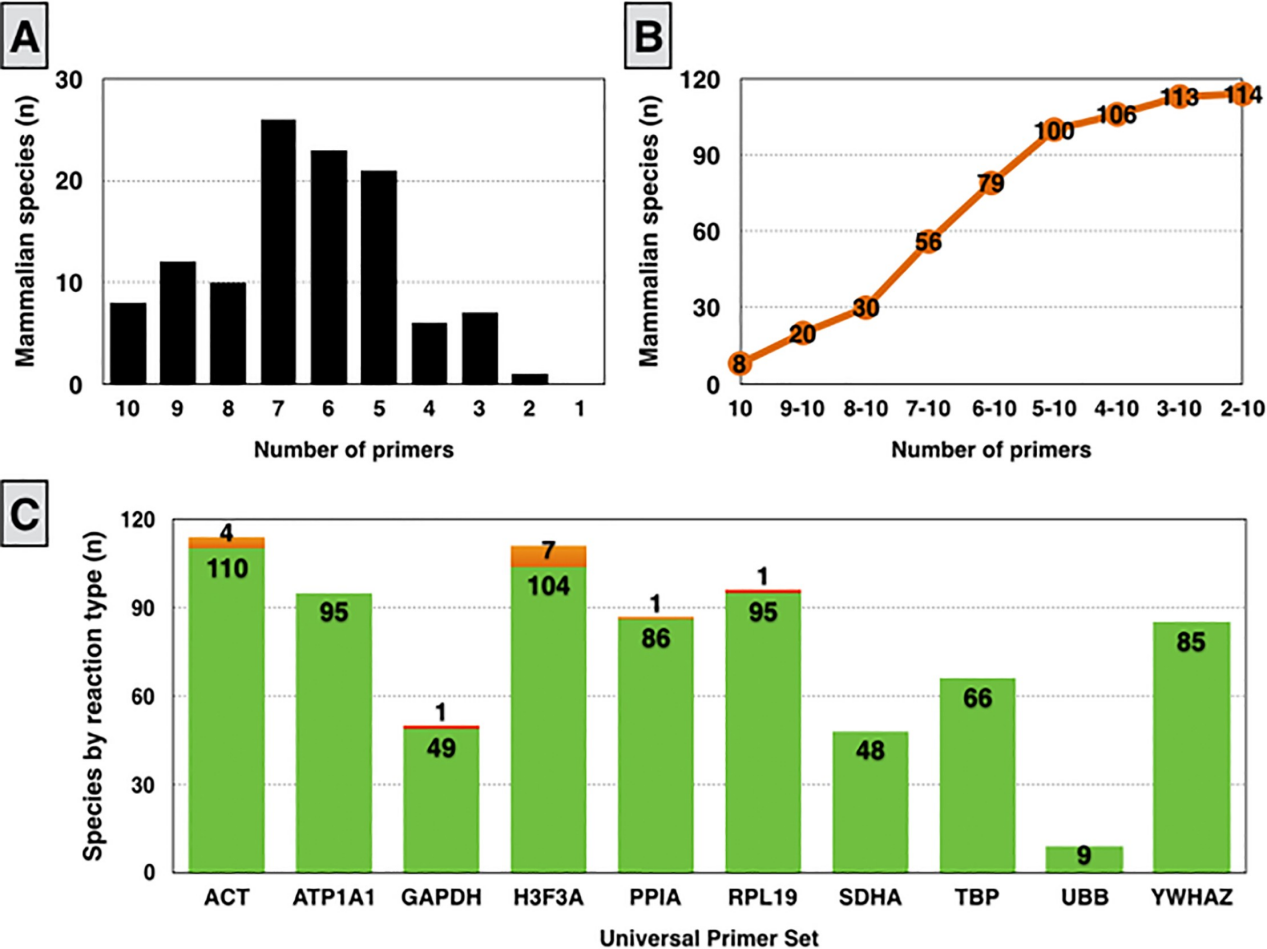
Multi-species primer set (MSPS) specificity in mammals. Number of species covered by varying primers, MSPS coverage by the number of primers, and primer-specific specificity.

A broader primer-BLAST search of each primer from the MSPS for mammalian species showed that they would amplify at least two gene products in 114 species, from 24 orders and 96 genera (S2 Table). A clear distinction is made from the number of species covered by a varying number of primers (Fig 6). Most species are covered by five to seven primers, followed by species attained by eight to ten primers (Fig 6). Only eight species are covered by all ten primers, but the number increases to 79 species for six or more primers (Fig 6).

Based on the Primer-BLAST search, the expected rate of positive specific reactions was 98.16% (749 of 763 potential reactions), with 1.57% (12 of 763) of non-specific reactions (i.e., gene transcript of interest plus other unintended transcripts), and 0.26% unspecific reactions (2 of 763 potential reactions). The *in silico* primer specificity among the MSPS in all retrieved mammalian species is expected to vary from 93.69% of *H3F3A* to 100.00% for several primers (*ATP1A1*, *SDHA*, *TBP*, *UBB*, and *YWHAZ*).

Most species found in the primer-BLAST search were representative of five orders (Artiodactyla, Rodentia, Primata, Carnivorae, and Chiroptera; S2 Table), thus representing 73.68% of the mammals. There were clear distinctions of primer specificity among orders and species (S6 Fig). As expected, most Artiodactyla species (ruminants) were covered by all primers (S2 Table), but with some species-specific exceptions, mostly for *UBB* (S6 Fig). Curiously, *Bos indicus* displayed polymorphisms that are predicted to not allow *SDHA* and *TBP* gene product amplifications, in contrast to *B*. *taurus* and *Bos mutus* (S2 Table). Several non-ruminant artiodactyla species were also covered by the MSPS as determined by primer-BLAST (S2 Table). Primate transcriptomes were mostly covered by six primers (*ACTIN*, *ATP1A1*, *H3F3A*, *PPIA*, *RPL19*, and *YWHAZ*), while rodents were generally covered by four (*ACTIN*, *ATP1A1*, *H3F3A*, and *TBP*), carnivores by eight (*ACTIN*, *ATP1A1*, *GAPDH*, *H3F3A*, *RPL19*, *SDHA*, *TBP*, and *YWHAZ*), and Chiroptera by four primers (*ACTIN*, *GAPDH*, *PPIA*, and *RPL19*). Finally, the assessment by primer-BLAST of other five non-mammalian species showed that one to three primers are expected to amplify gene transcripts (S3 Table).

## Discussion

Gene expression analysis has been a fruitful tool for understanding the establishment and maintenance of cellular states [1–2]. However, less attention has been drawn to RT-qPCR conditions, further leading to data of lower reproducibility [8,14,19]. Within the requirements of reliable RT-qPCR, RG selection remains paramount and must be carried out for each experimental setup [13–18].

The strategy of designing primers at conversed sequences for selected livestock species allowed the development of a MSPS for potential use in the RT-qPCR assay. Although “universal” primers have been described in different contexts [30–33], to the best of our knowledge, a MSPS remained to be described for RT-qPCR applicable to multiple order-specific species and for inter-genus gene expression analysis. There are several evidences that the here described MSPS is useful for the livestock species; firstly, all ten primers detected as single amplicons by conventional RT-PCR in four different genera. Secondly, all ten primers efficient in *O*. *aries—B*. *taurus* bulk cDNA, *O*.*aries* and *B*. *taurus* fibroblast cDNA or *O*. *aries* cumulus cDNA, suggesting that primer efficiency was dependent upon cell-type specific gene transcript abundance. Furthermore, the multi-species mixed cDNA approach has been validated under other experimental contexts [37,41]. Thirdly, RT-qPCR normalization was attained in *B*. *taurus—O*. *aries* inter-genus RT-qPCR as assessed by five algorithms. Fourthly, some primers have been used for intra-specific RT-PCR in eggs and early embryos of livestock species [42,43].

Different RG sets were found for *O*. *aries*—*B*. *taurus* inter-genus RT-qPCR-based relative gene expression normalization using three algorithms. The GeNorm normalization using RPL19 and YWHAZ showed a substantial difference to the NormFinder ATP1A1 and UBB-pairwise index, most likely due to methodological differences. However, the addition of the third and fourth RGs to the GeNorm RG pair demonstrated a parallel between normalization software packages. Both methods (delta-CT and RefFinder) generated similar RG rankings but did not deviate far from the other aforementioned algorithms. Numerous strategies have been initially described for identifying a single RG [18], and later the RG index approach was introduced for more accurate RT-qPCR data normalization [21,26]. Similar reports for RG validation used various numbers of candidate gene transcripts and algorithms [13–17,44]. More importantly, some reports have found similar RG sets using different software packages [45], while other have reported contrasting RG sets [46], particularly using the most widely used algorithms [21,26,44].

The widely used *ACT* and *GAPDH* genes proved to be within the most unstable ones, as it is becoming evident in many other experimental conditions and organisms [27,47], further highlighting the importance of careful and unbiased RG selection [21–26]. Novel not yet tested candidate RG have also proven to be more stable than the widely used ones [47], at least under some circumstances. The critical selection of candidate RG also has the capability to diminish or avoid potential selection of co-regulated RGs, which may weaken RT-qPCR data normalization [48]. Primer efficiency variation is another critical experimental parameter that may affect RT-qPCR data normalization [48], as observed for the gene of choice TLR4, as described above Nonetheless, no primer efficiency variation was found, since RG primer efficiency was attained using single-species or bulked cDNA of *B*. *taurus—O*. *aries* from fibroblasts, as previously described elsewhere [37,38]. Despite the fact that algorithms indicated different RG indexes or rankings, it became clear that all results from such software packages converged to similar results. Collectively, these findings demonstrate that different RG indexes can be successfully used for inter-genus RT-qPCR data normalization. Further evidence suggests that the retrieved RG described above may indeed be stable across other tissues, as demonstrated by RNA-Seq data from seven somatic tissues.

Both *TRL4* and *ZFX* candidate genes were up-regulated in *B*. *taurus* fibroblasts in comparison to their *O*. *aries* counterparts. Toll-like receptors (TLRs) are transmembrane-spanning proteins and are part of the innate immune response, displaying high species-specific, tissue-specific, and individual variation in transcript availability [49,50], thus reinforcing the results described above. The *ZFX* is an X-linked gene that encodes a transcription factor expressed throughout development [51–53]. Since *ZFX* is a highly conserved protein across mammals [54], differences in transcript availability between *O*. *aries* and *B*. *taurus* are possibly caused by differences in cis-regulatory sequences or varying trans-activating proteins. Gene expression profiling across species is an instructive tool for understanding species-specific gene expression signatures [3,5]. However, less attention has been drawn to the quantitative measurements of gene expression among species [13,16], particularly in a direct, unbiased fashion [55,56]. Collectively, these results suggest that fibroblasts may holdspecies-specific gene stoichiometry as described in other systems [57,58], and thus may have biological significance as for cell potency, lineage specification, and cellular reprogramming [57–59]. However, further evidence is required for full appreciation of this variation at transcriptomic level across species. Alternatively, the MSPS described here could also be used for other applications such as diagnostics [60], genetic characterization [61], nucleus-cytoplasm interactions [62], and interspecies cell-fusion or nuclear transfer experiments [56,63].

An *in silico* search in mammals using primer-BLAST showed that MSPS is predicted to amplify at least two gene transcripts of over a hundred species. More importantly, a total of around eighty species would be covered by six or more primers from the MSPS. These results are striking by the fact that 24 mammalian orders were represented in the initial *in silico* screening; with around half of such species are rodents or primates, including many common animal models for developmental biology and biomedical research (e.g., rat, rhesus, and gorilla). The MSPS can also be improved for order-specific RT-qPCR by the design of a small subset of primers. Additionally, the results reinforce that the potential of the MSPS has been underestimated and could be potentially extended to other classes of gene transcripts for cross-species or inter-genus gene expression profiling via RT-qPCR. In sum, the design of a multi-species primer set allows for inter-genus gene expression analysis between *O*. *aries* and *B*. *taurus* primary cells and further reveals species-specific gene transcript abundance of key transcription factors.

## Supporting information

S1 Table Primer efficiency, coefficient correlation, slope, and Y-intercept derived from the standard curve of each candidate reference gene from the multi-species primer set (MSPS) using *O*. *aries* cumulus cDNA via RT-qPCR assay. (PDF)

S2 Table *In silico* analysis of multi-species primer set (MSPS) specificity in mammalian orders determined by primer-BLAST. (PDF)

S3 Table *In silico* analysis of multi-species primer set (MSPS) specificity in selected non-mammalian species determined by primer-BLAST. (PDF)

S4 Table Primer efficiency, coefficient correlation, slope, and Y-intercept derived from the standard curve of each candidate reference gene from the multi-species primer set (MSPS) using non-mixed *O*. *aries* and *B*. *taurus* fibroblast cDNA via RT-qPCR assay. (DOCX)

S1 Fig Description of cDNA type used for primer efficiency testing and relative gene expression analysis. (TIFF)

S2 Fig Total RNA extraction efficiency and RNA 280/260 and 260/230 ratios from somatic cell samples of selected livestock species (*B*. *taurus*, *B*. *bubalis*, *C*. *hircus*, and *O*. *aries*) investigated in the experiment. (TIFF)

S3 Fig Experimental design (A), site of primer annealing in gene transcripts (B), and number of gene transcripts covered by the multi-species primer set (MSPS) in selected livestock species (*B*. *taurus*, *B*. *bubalis*, *C*. *hircus*, and *O*. *aries*).
(TIFF)

S4 Fig Detection of gene transcripts (melting curves) from the multi-species primer set (MSPS) in selected livestock species by quantitative reverse transcription PCR (RT-qPCR). (PDF)

S5 Fig Relative expression of candidate reference genes in seven tissues of *B*. *taurus* and *O*. *aries* as determined by the gene expression atlas at the European Bioinformatics Institute website (https://www.ebi.ac.uk/gxa/home). TPM: transcripts per million.
(TIFF)

S6 Fig Order-specific multi-species primer set (MSPS) specificity in mammals. Number of species covered by varying primers, MSPS coverage by the number of primers, and primer-specific specificity.
(TIFF)
