# Supplementary material for: Inter-genus gene expression analysis in livestock fibroblasts using reference gene validation based upon a multi-species primer set
Source: PLoS One. 2019 Aug 14;14(8):e0221170. doi: 10.1371/journal.pone.0221170 (PMC6693880; doi:10.1371/journal.pone.0221170)
Supplement: S1 Table — (PDF) [file pone.0221170.s001.pdf]

S1 Table. Primer efficiency, coefficient correlation, slope, and Y intercept derived from the standard curve of each candidate reference gene from the universal primer set using *Ovis aries* cumulus cDNA via RT-qPCR assay.

Gene symbol*	E (%)	NTC (Cq)	Correlation Coefficient (*R*)	Slope	Y intercept
**ACT**	**97.53**	**33.97**	**-0.998**	**-3.38**	**25.82**
**ATP1A1**	**103.20**	**-**	**-0.998**	**-3.25**	**28.72**
**GAPDH**	**90.69**	**33.39**	**-0.998**	**-3.57**	**28.88**
**PPIA**	**101.02**	**-**	**-0.999**	**-3.30**	**24.74**
**RPL19**	**103.05**	**-**	**-0.996**	**-3.25**	**25.01**
**SDHA**	**94.42**	**-**	**-0.996**	**-3.46**	**30.95**
**TBP**	**110.76**	**32.88**	**-0.995**	**-3.09**	**31.83**
**UBB**	**96.88**	**-**	**-0.998**	**-3.40**	**29.26**
**YWHAZ**	**103.07**	**-**	**-0.995**	**-3.25**	**28.31**

*Actin (ACT), ATPase Na+/K+ transporting subunit alpha 1 (ATP1A1), Glyceraldehyde 3-phosphate dehydrogenase (GAPDH), H3 histone, family 3A (H3F3A), Peptidylprolyl isomerase A (PPIA), Ribosomal protein L19 (RPL19), Succinate dehydrogenase complex flavoprotein subunit A (SDHA), TATA-binding protein (TBP), Ubiquitin B (UBB), Tyrosine 3 - monooxygenase / tryptophan 5 - monooxygenase activation protein zeta (YWHAZ). Cq: Cycle of quantification. E: efficiency. N.T.C.: No template control.
