# Supplementary material for: Inter-genus gene expression analysis in livestock fibroblasts using reference gene validation based upon a multi-species primer set
Source: PLoS One. 2019 Aug 14;14(8):e0221170. doi: 10.1371/journal.pone.0221170 (PMC6693880; doi:10.1371/journal.pone.0221170)
Supplement: S2 Table — (PDF) [file pone.0221170.s002.pdf]

S2 Table. *In silico* analysis of universal primer set specificity in mammalian orders determined by primer-BLAST. Predicted specific reactions (Y - Green), not expected to amplify (N), unspecific reactions (U - red), and simultaneous predicted specific and unspecific reactions (Y - orange). Actin (ACT), ATPase Na+/K+ transporting subunit alpha 1 (ATP1A1), Glyceraldehyde 3-phosphate dehydrogenase (GAPDH), H3 histone, family 3A (H3F3A), Peptidylprolyl isomerase A (PPIA), Ribosomal protein L19 (RPL19), Succinate dehydrogenase complex flavoprotein subunit A (SDHA), TATA-binding protein (TBP), Ubiquitin B (UBB), Tyrosine 3 - monooxygenase / tryptophan 5 - monooxygenase activation protein zeta (YWHAZ).

	SPECIES	ACT	ATP1A1	GAPDH	H3F3A	PPIA	RPL19	SDHA	TBP	UBB	YWHAZ
Order artidatylia	Bison bison bison	Y	Y	Y	Y	Y	Y	Y	Y	Y	Y
	Bos indicus	Y	Y	Y	Y	Y	Y	N	N	Y	Y
	Bos mutus	Y	Y	Y	Y	Y	Y	Y	Y	Y	Y
	Bos taurus	Y	Y	Y	Y	Y	Y	Y	Y	Y	Y
	Bubalus bubalis	Y	Y	Y	Y	Y	Y	Y	Y	Y	Y
	Camelus bactrianus	Y	Y	Y	Y	Y	Y	Y	Y	N	Y
	Camelus dromedarius	Y	Y	Y	Y	Y	Y	Y	Y	N	Y
	Camelus ferus	Y	Y	Y	Y	Y	Y	Y	Y	N	N
	Capra hircus	Y	Y	Y	Y	Y	Y	Y	Y	Y	Y
	Odocoileus virginianus texanus	Y	Y	Y	Y	Y	Y	Y	Y	Y	Y
	Ovis aries	Y	Y	Y	Y	Y	Y	Y	Y	Y	Y
	Ovis aries musimon	Y	N	Y	Y	Y	Y	Y	Y	N	N
	Pantholops hodgsonii	Y	Y	Y	Y	Y	Y	Y	Y	Y	Y
	Sus scrofa	Y	Y	Y	Y	Y	Y	Y	Y	N	Y
	Vicugna pacos	Y	Y	Y	Y	Y	Y	Y	Y	N	Y

	SPECIES	ACT	ATP1A1	GAPDH	H3F3A	PPIA	RPL19	SDHA	TBP	UBB	YWHAZ
Order carnivorae	Acinonyx jubatus	Y	Y	Y	Y	N	Y	Y	Y	N	N
	Ailuropoda melanouleuca	Y	Y	N	N	N	N	N	N	N	N
	Canis lupus familiaris	Y	Y	Y	Y	Y	Y	Y	Y	N	Y
	Enhydra lutris	Y	Y	Y	Y	N	N	Y	Y	N	Y
	Felis catus	Y	Y	Y	Y	N	Y	Y	Y	N	Y
	Leptonychotes weddellii	Y	Y	Y	Y	N	Y	N	Y	N	Y
	Mustela putorius furo	Y	Y	Y	Y	Y	N	Y	Y	N	Y
	Neomonachus schauinslandi	Y	Y	Y	Y	Y	Y	Y	Y	N	Y
	Odobenus rosmarus divergens	Y	Y	Y	Y	Y	Y	Y	Y	N	Y
	Panthera pardus	Y	Y	Y	Y	Y	Y	Y	Y	N	Y
	Panthera tigris	Y	Y	Y	Y	Y	Y	Y	Y	N	Y
	Ursus maritimus	Y	Y	Y	Y	N	Y	Y	Y	N	N

	SPECIES	ACT	ATP1A1	GAPDH	H3F3A	PPIA	RPL19	SDHA	TBP	UBB	YWHAZ
Order chiroptera	Desmodus rotundos	Y	Y	Y	Y	N	Y	Y	N	N	Y
	Eptesicus fuscus	Y	Y	Y	Y	N	Y	N	Y	N	Y
	Hipposideros armiger	Y	Y	Y	Y	Y	Y	N	N	N	N
	Miniopterus natalensis	Y	Y	Y	Y	N	Y	Y	N	N	Y
	Myotis brandtii	Y	N	Y	Y	Y	Y	N	Y	N	Y
	Myotis davidii	Y	N	Y	Y	Y	Y	N	Y	N	N
	Myotis lucifugus	Y	N	Y	N	Y	Y	N	Y	N	N
	Pteropus alecto	Y	Y	N	Y	Y	Y	Y	N	N	Y
	Pteropus vampyrus	Y	N	N	Y	Y	Y	Y	N	N	Y
	Rhinolophus sinicus	Y	Y	Y	Y	Y	Y	N	N	N	N
	Rousettus aegyptiacus	Y	N	N	Y	Y	Y	Y	Y	N	Y

Table S2. *In silico* analysis of universal primer set specificity in mammalian orders determined by primer-BLAST. Predicted specific reactions (Y - Green), not expected to amplify (N), unspecific reactions (U - red), and simultaneous predicted specific and unspecific reactions (Y - orange). (**Continued**).

	SPECIES	ACT	ATP1A1	GAPDH	H3F3A	PPIA	RPL19	SDHA	TBP	UBB	YWHAZ
Order primata	Aotus nancymaae rotundus	Y	Y	Y	Y	Y	Y	N	N	N	Y
	Callithrix jacchus	Y	Y	N	Y	Y	Y	N	N	N	Y
	Carlito syrichta	Y	Y	U	Y	Y	Y	N	Y	N	N
	Cebus capucinus imitator	Y	Y	Y	Y	Y	Y	N	N	N	Y
	Cercocebus atys	Y	Y	N	Y	Y	Y	Y	N	N	Y
	Chlorocebus sabaeus	Y	Y	N	Y	Y	Y	Y	N	N	Y
	Colobus angolensis palliatus	Y	Y	N	Y	Y	Y	N	N	N	Y
	Gorilla gorilla gorilla	Y	Y	N	Y	Y	Y	N	N	N	Y
	Homo sapiens	Y	Y	N	Y	Y	Y	N	N	N	Y
	Macaca Fascicularis	Y	Y	N	Y	Y	Y	Y	N	N	Y
	Macaca mulatta	Y	Y	N	Y	Y	Y	Y	N	N	Y
	Macaca nemestrina	Y	Y	N	Y	Y	Y	Y	N	N	Y
	Mandrillus leucophaeus	Y	Y	N	Y	Y	Y	Y	N	N	N
	Microcebus murinus	Y	Y	N	Y	Y	Y	N	Y	N	Y
	Nomascus leucogenys	Y	Y	N	Y	Y	Y	N	N	N	Y
	Otolemur garnettii	Y	Y	Y	Y	Y	N	N	Y	N	Y
	Pan paniscus	Y	Y	N	Y	Y	Y	N	N	N	N
	Pan troglodytes	Y	Y	N	Y	Y	Y	N	N	N	Y
	Papio anubis	Y	Y	N	Y	Y	Y	N	N	N	Y
	Piliocolobus tephrosceles	Y	Y	N	Y	Y	Y	N	N	N	Y
	Pongo abelii	Y	Y	N	Y	Y	Y	N	N	N	Y
	Propithecus coquereli	Y	Y	N	Y	Y	Y	N	Y	N	Y
	Rhinopithecus bieti	Y	Y	N	Y	Y	Y	N	N	N	Y
	Rhinopithecus roxellana	Y	Y	Y	Y	Y	Y	Y	N	N	Y
	Saimiri boliviensis boliviensis	Y	Y	Y	Y	Y	Y	N	N	N	N

	SPECIES	ACT	ATP1A1	GAPDH	H3F3A	PPIA	RPL19	SDHA	TBP	UBB	YWHAZ
Order rodentia	Castor canadensis	Y	N	N	Y	Y	Y	N	Y	N	N
	Cavia porcellus	Y	N	N	Y	N	Y	N	Y	N	N
	Chinchilla lanigera	Y	Y	N	Y	N	N	N	Y	N	N
	Cricetulus griseus	Y	Y	N	Y	N	N	N	N	N	N
	Dipodomys ordii	Y	Y	N	Y	Y	Y	N	N	N	N
	Fukomys damarensis	Y	N	N	Y	Y	N	N	Y	N	N
	Heterocephalus glaber	Y	N	N	Y	N	N	N	Y	N	N
	Ictidomys tridecemlineatus	Y	Y	N	Y	Y	Y	N	N	N	Y
	Jaculus jaculus	Y	Y	N	Y	N	U	N	N	N	Y
	Marmota marmota	Y	Y	N	Y	Y	Y	Y	Y	N	N
	Meriones unguiculatus	Y	N	N	Y	Y	N	N	Y	N	Y
	Mesocricetus auratus	Y	Y	Y	Y	N	Y	N	Y	N	N
	Microtus ochrogaster	Y	Y	N	Y	Y	Y	N	Y	N	Y
	Mus caroli	Y	Y	N	Y	Y	N	N	Y	N	Y
	Mus musculus	Y	Y	N	Y	Y	N	N	Y	N	Y
	Mus pahari	Y	Y	N	Y	N	N	N	Y	N	N
	Nannospalax galili	Y	N	N	Y	Y	Y	N	N	N	Y
	Octodon degus	Y	Y	Y	Y	N	Y	N	Y	N	N
	Octodon unguiculatus	Y	N	N	Y	Y	N	N	Y	N	Y
	Peromyscus maniculatus bairdii	Y	Y	N	Y	Y	Y	N	Y	N	Y
	Rattus norvegicus	Y	Y	N	Y	Y	N	N	Y	N	Y

Table S2. *In silico* analysis of universal primer set specificity in mammalian orders determined by primer-BLAST. Predicted specific reactions (Y - Green), not expected to amplify (N), unspecific reactions (U - red), and simultaneous predicted specific and unspecific reactions (Y - orange). **(Continued).**

	SPECIES	ACT	ATP1A1	GAPDH	H3F3A	PPIA	RPL19	SDHA	TBP	UBB	YWHAZ
Other Orders	Balaenoptera acutorostrata scammoni	Y	Y	N	Y	Y	Y	Y	Y	N	Y
	Ceratotherium simum simum	Y	Y	N	Y	Y	Y	N	Y	N	Y
	Chrysochloris asiatica	Y	Y	Y	Y	N	Y	N	N	N	Y
	Condylura cristata	Y	Y	N	Y	Y	Y	N	N	N	Y
	Dasypus novemcinctus	Y	Y	Y	N	Y	Y	N	N	N	Y
	Delphinapterus leucas	Y	Y	N	Y	Y	Y	Y	Y	N	Y
	Echinops telfairi	Y	Y	N	Y	N	Y	N	Y	N	N
	Elephantulus edwardii	Y	Y	N	Y	Y	Y	N	N	N	Y
	Equus asinus	Y	Y	Y	Y	Y	Y	Y	Y	N	Y
	Equus caballus	Y	Y	Y	Y	Y	Y	Y	Y	N	Y
	Equus przewalskii	Y	Y	Y	Y	Y	Y	Y	Y	N	Y
	Erinaceus europaeus	Y	N	N	Y	N	N	Y	N	N	Y
	Galeopterus variegatus	Y	Y	Y	Y	Y	Y	N	Y	N	N
	Lipotes vexillifer	Y	N	N	Y	Y	Y	Y	Y	N	Y
	Loxodonta africana	Y	Y	Y	Y	Y	Y	N	N	N	Y
	Manis javanica	Y	Y	Y	Y	Y	Y	Y	Y	N	Y
	Monodelphis domestica	Y	N	N	Y	N	Y	N	N	N	N
	Neophocaena asiaeorientalis asiaeorientalis	Y	Y	N	Y	Y	Y	Y	Y	N	Y
	Ochotona princeps	Y	Y	N	Y	N	N	N	Y	N	Y
	Orcinus orca	Y	Y	N	Y	Y	Y	Y	Y	N	Y
	Ornithorhynchus anatinus	Y	N	N	Y	N	Y	N	N	N	N
	Orycteropus afer afer	Y	Y	N	Y	N	N	N	N	N	Y
	Oryctolagus cuniculus	Y	Y	N	Y	N	Y	N	Y	N	Y
	Phascolarctos cinereus	Y	N	N	Y	N	N	N	N	N	Y
	Physeter catodon	Y	Y	N	Y	Y	Y	N	Y	N	Y
	Sarcophilus harrisii	Y	N	N	Y	N	N	N	N	N	Y
	Sorex araneus	Y	Y	N	Y	Y	Y	N	N	N	N
	Trichechus manatus latirostris	Y	Y	Y	Y	Y	Y	N	N	N	Y
	Tupaia chinensis	Y	Y	N	Y	Y	Y	N	Y	N	Y
	Tursiops truncatus	Y	Y	N	Y	Y	Y	Y	N	N	Y
