# Supplementary material for: Inter-genus gene expression analysis in livestock fibroblasts using reference gene validation based upon a multi-species primer set
Source: PLoS One. 2019 Aug 14;14(8):e0221170. doi: 10.1371/journal.pone.0221170 (PMC6693880; doi:10.1371/journal.pone.0221170)
Supplement: S3 Table — (PDF) [file pone.0221170.s003.pdf]

Table S3. *In silico* analysis of universal primer set specificity in non-mammalian model organism determined by primer-BLAST. Predicted specific reactions (Y - Green) and not expected to amplify (N).

	ACT	ATP1A1	GAPDH	H3F3A	PPIA	RPL19	SDHA	TBP	UBB	YWHAZ
Anolis carolinensis	Y	N	Y	N	N	N	N	N	N	N
Drosophila melanogater	Y	N	N	N	N	N	N	N	N	N
Danio rerio	Y	Y	N	N	N	N	N	N	N	N
Gallus gallus	Y	N	N	Y	N	N	N	N	N	N
Xenopus laevis	Y	N	N	Y	Y	N	N	N	N	N

Actin (ACT), ATPase Na+/K+ transporting subunit alpha 1 (ATP1A1), Glyceraldehyde 3-phosphate dehydrogenase (GAPDH), H3 histone, family 3A (H3F3A), Peptidylprolyl isomerase A (PPIA), Ribosomal protein L19 (RPL19), Succinate dehydrogenase complex flavoprotein subunit A (SDHA), TATA-binding protein (TBP), Ubiquitin B (UBB), Tyrosine 3 - monooxygenase / tryptophan 5 - monooxygenase activation protein zeta (YWHAZ).
