# Supplementary material for: Inter-genus gene expression analysis in livestock fibroblasts using reference gene validation based upon a multi-species primer set
Source: PLoS One. 2019 Aug 14;14(8):e0221170. doi: 10.1371/journal.pone.0221170 (PMC6693880; doi:10.1371/journal.pone.0221170)
Supplement: S4 Table — (DOCX) [file pone.0221170.s004.docx]

**S4 Table.** Primer efficiency, coefficient correlation, slope, and Y intercept derived from the standard curve of each candidate reference gene from the multi-species primer set using *O. aries* or *B. taurus* fibroblast cDNA via RT-qPCR assay.

| **Gene** | **Species** | **E (%)** | **NTC (Cq)** | **Correlation Coefficient (R)** | **Slope** | **Y intercept** | **Error** |
| --- | --- | --- | --- | --- | --- | --- | --- |
| **ACT** | *O. aries* | 97.53 | - | -0.998 | - 3.38 | 25.82 | 0.013 |
|  | *B. taurus* | 99.09 | - | -0.998 | - 3.34 | 29.87 | 0.014 |
| **GAPDH** | *O. aries* | 90.69 | - | -0.998 | - 3.57 | 28.88 | 0.014 |
|  | *B. taurus* | 102.04 | - | -0.993 | - 3.27 | 31.69 | 0.019 |
| **PPIA** | *O. aries* | 101.02 | - | -0.999 | - 3.30 | 24.74 | 0.012 |
|  | *B. taurus* | 99.21 | - | -0.997 | - 3.34 | 29.71 | 0.015 |
| **RPL19** | *O. aries* | 103.05 | - | -0.996 | - 3.25 | 25.01 | 0.019 |
|  | *B. taurus* | 99.52 | - | -0.969 | - 3.33 | 27.42 | 0.058 |
| **YWHAZ** | *O. aries* | 103.07 | - | -0.995 | - 3.25 | 28.31 | 0.021 |
|  | *B. taurus* | 92.26 | - | -0.990 | - 3.52 | 32.59 | 0.025 |

Actin (Act), Glyceraldehyde 3-phosphate dehydrogenase (GAPDH), Peptidylprolyl isomerase A (PPIA), Ribosomal protein L19 (RPL19), Tyrosine 3 - monooxygenase / tryptophan 5 - monooxygenase activation protein zeta (YWHAZ). Cq: Cycle of quantification. E: efficiency. N.T.C.: No template control.
