# Supplementary figures and images for: Inter-genus gene expression analysis in livestock fibroblasts using reference gene validation based upon a multi-species primer set
Source: PLoS One. 2019 Aug 14;14(8):e0221170. doi: 10.1371/journal.pone.0221170 (PMC6693880; doi:10.1371/journal.pone.0221170)

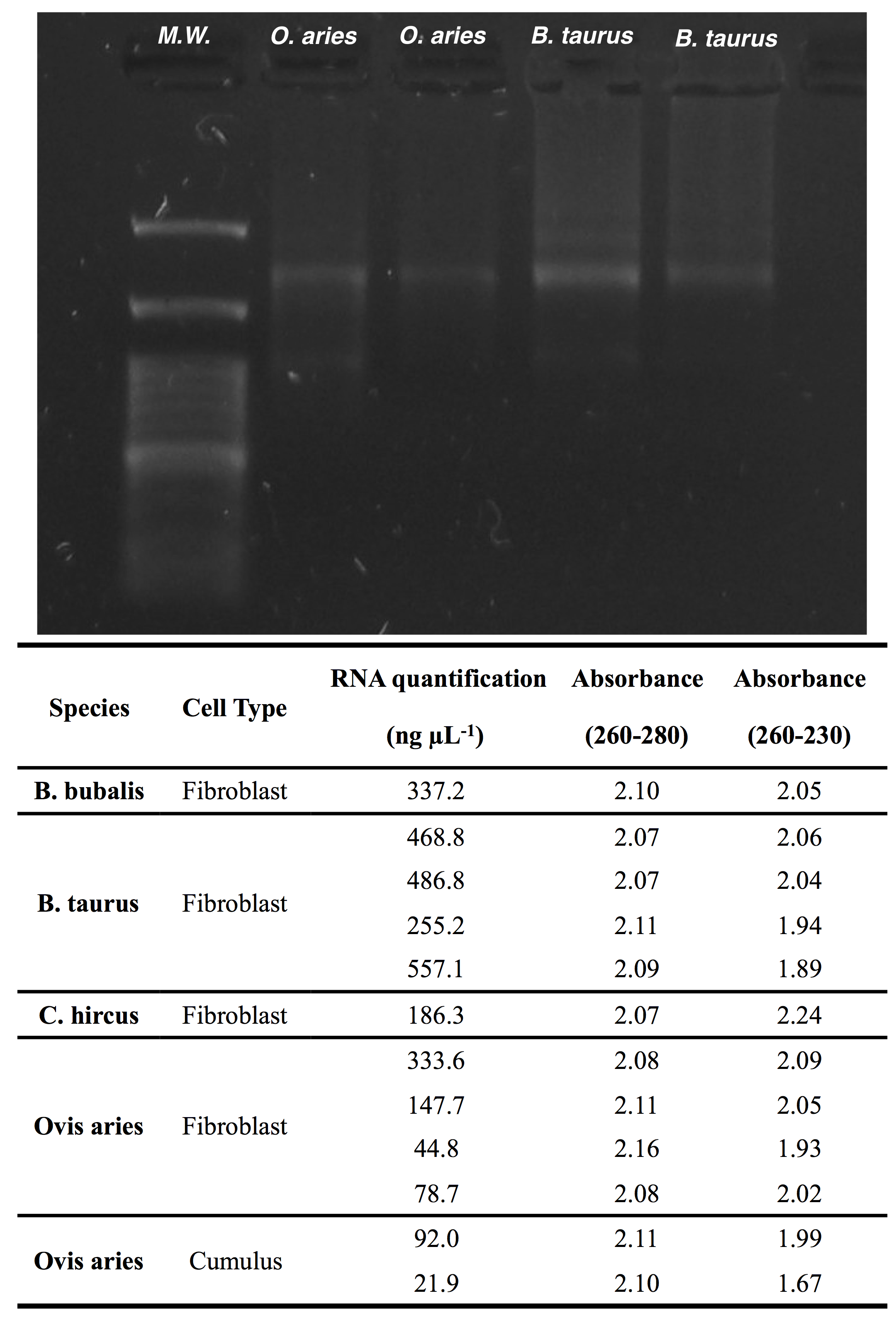

Supplement: S1 Fig — (TIFF) [file pone.0221170.s005.tiff]

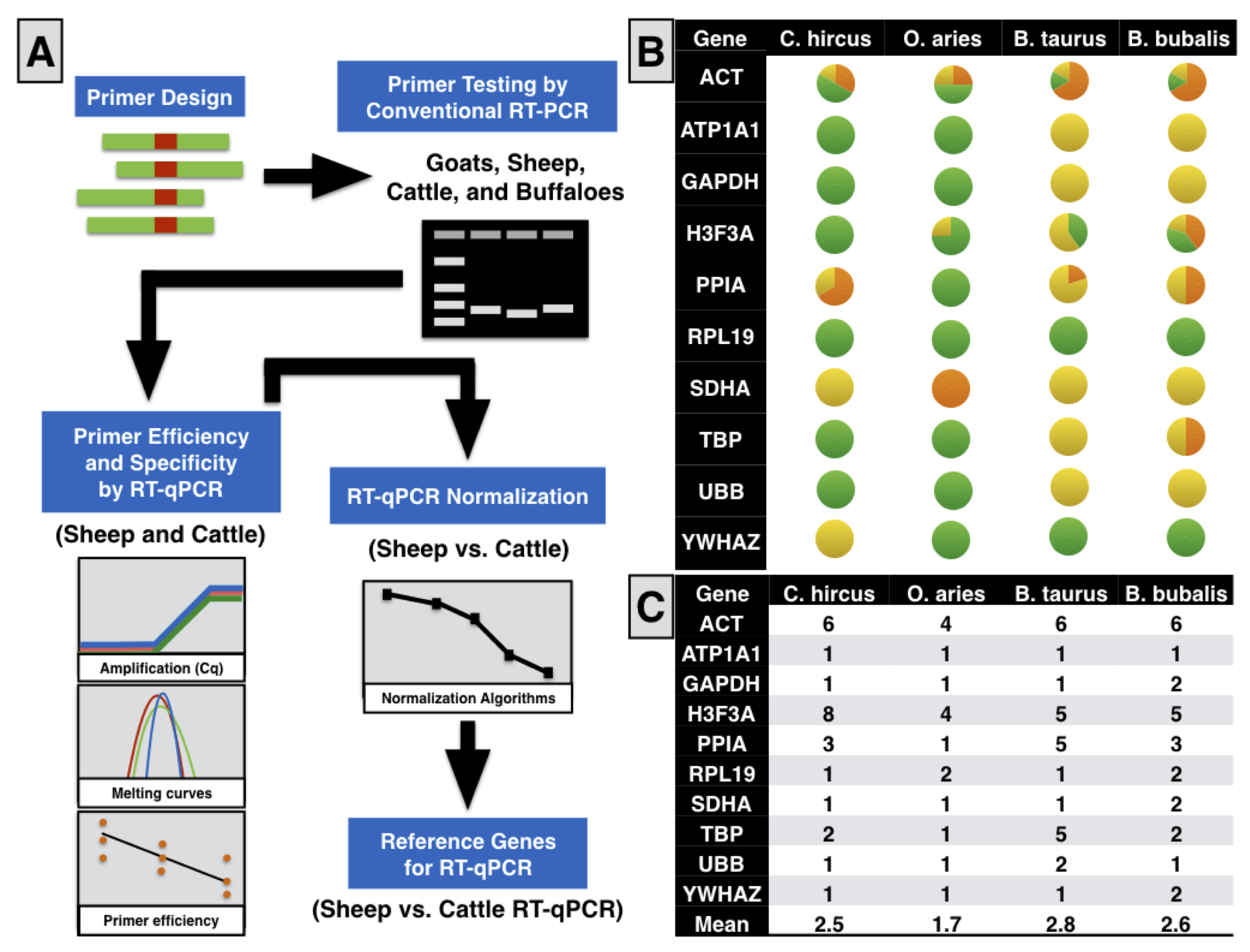

Supplement: S2 Fig — (TIFF) [file pone.0221170.s006.tiff]

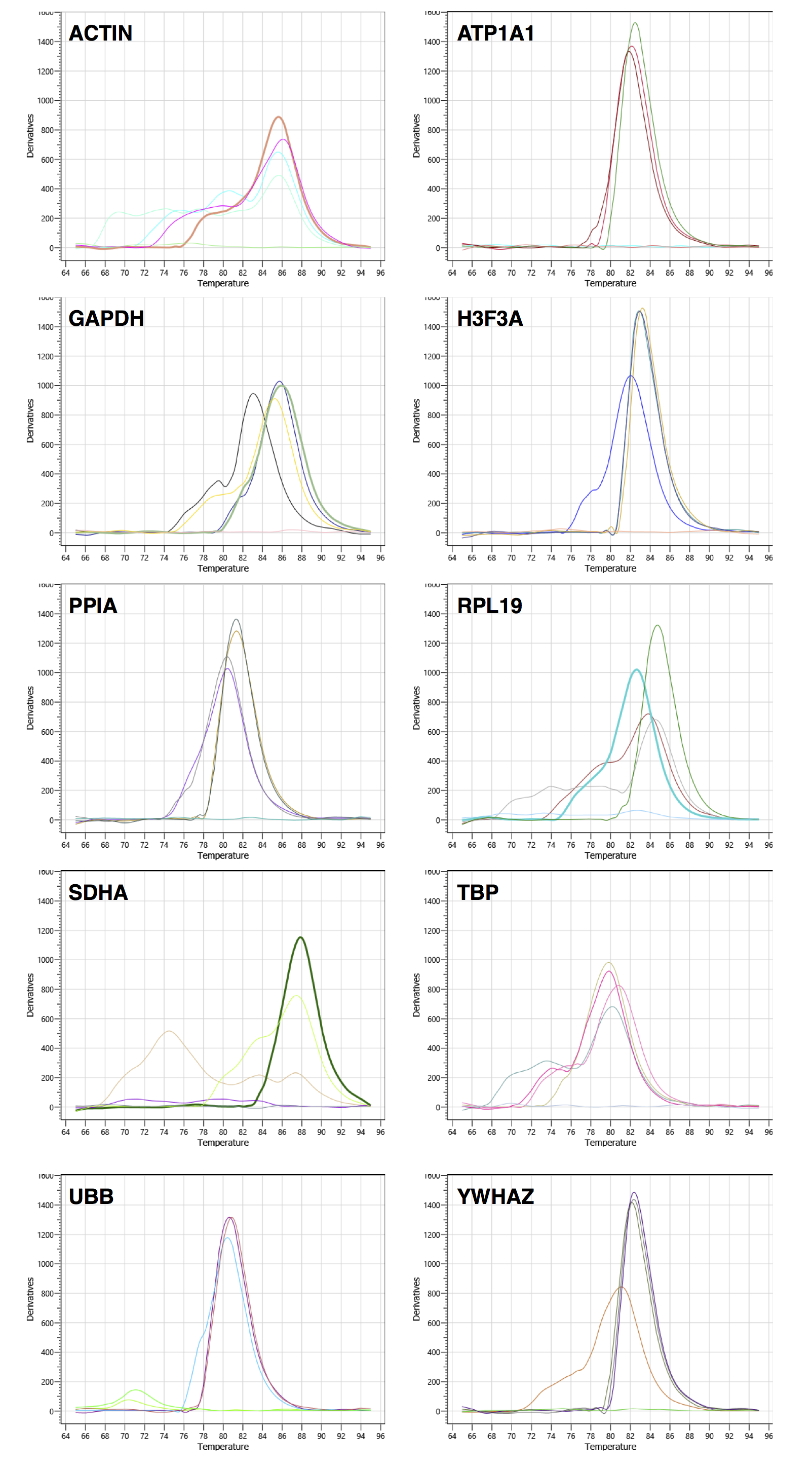

Supplement: S3 Fig — Experimental design (A), site of primer annealing in gene transcripts (B), and number of gene transcripts covered by the multi-species primer set (MSPS) in selected livestock species (B. taurus, B. bubalis, C. hircus, and O. aries). (TIFF) [file pone.0221170.s007.tiff]

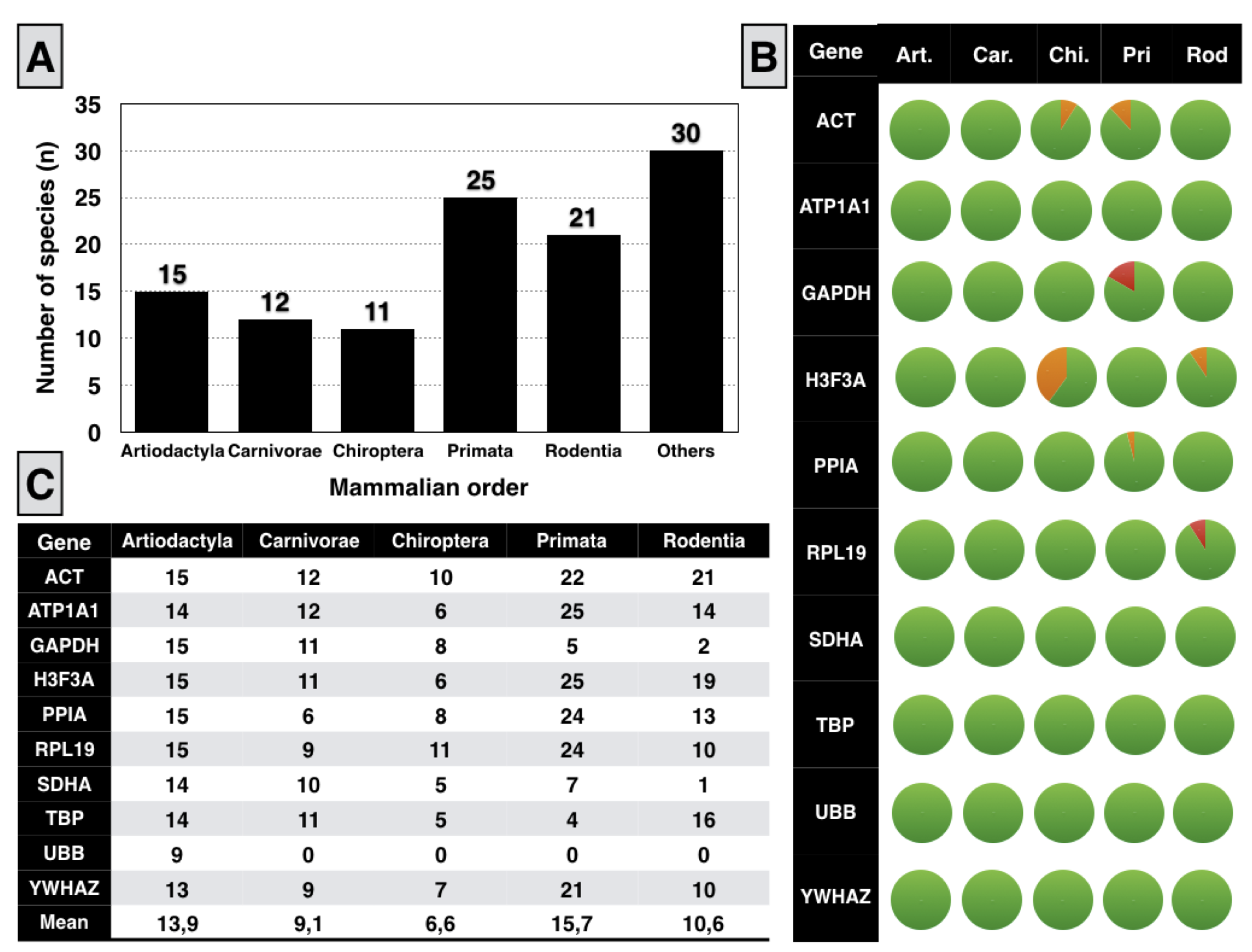

Supplement: S5 Fig — TPM: transcripts per million. (TIFF) [file pone.0221170.s009.tiff]

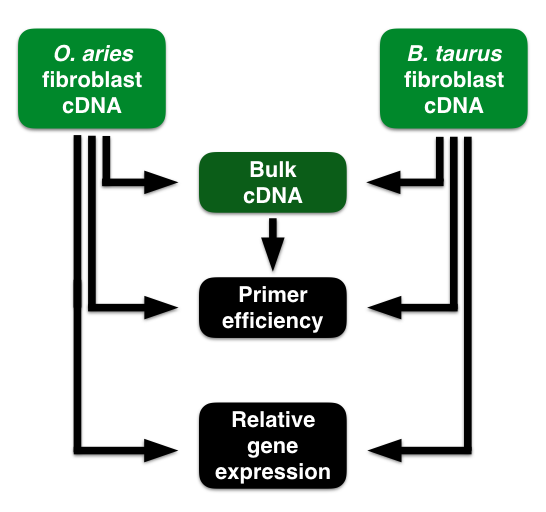

Supplement: S6 Fig — Number of species covered by varying primers, MSPS coverage by the number of primers, and primer-specific specificity. (TIFF) [file pone.0221170.s010.tiff]
