# Supplementary material for: Inter-genus gene expression analysis in livestock fibroblasts using reference gene validation based upon a multi-species primer set
Source: PLoS One. 2019 Aug 14;14(8):e0221170. doi: 10.1371/journal.pone.0221170 (PMC6693880; doi:10.1371/journal.pone.0221170)
Supplement: S4 Fig — (PDF) [file pone.0221170.s008.pdf]

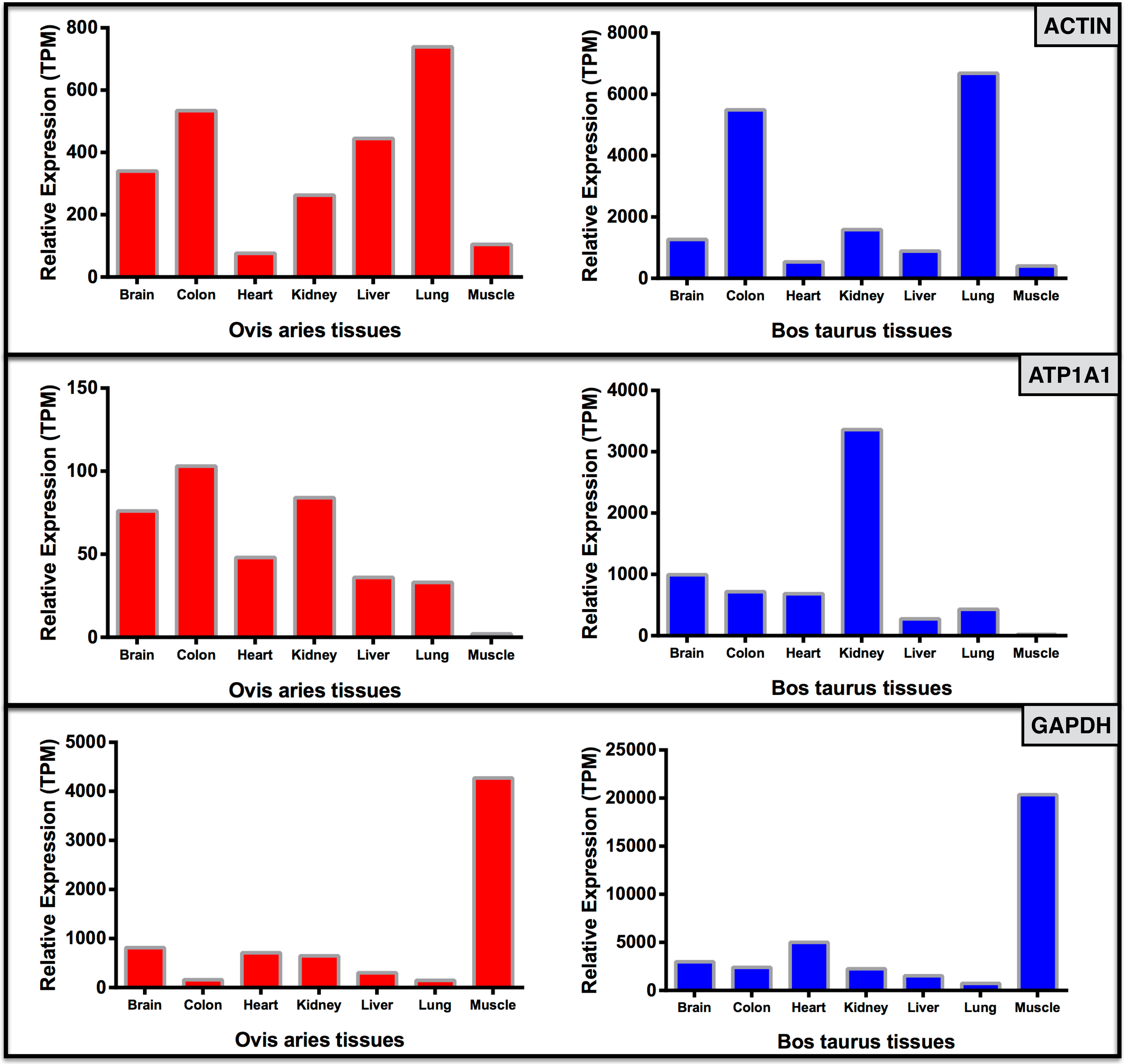
ACTIN
ATP1A1
GAPDH
Relative Expression (TPM)
Ovis aries tissues
Bos taurus tissues
Brain
Colon
Heart
Kidney
Liver
Lung
Muscle
0
200
400
600
800
2000
4000
6000
8000
50
100
150
1000
3000
5000
10000
15000
20000
25000

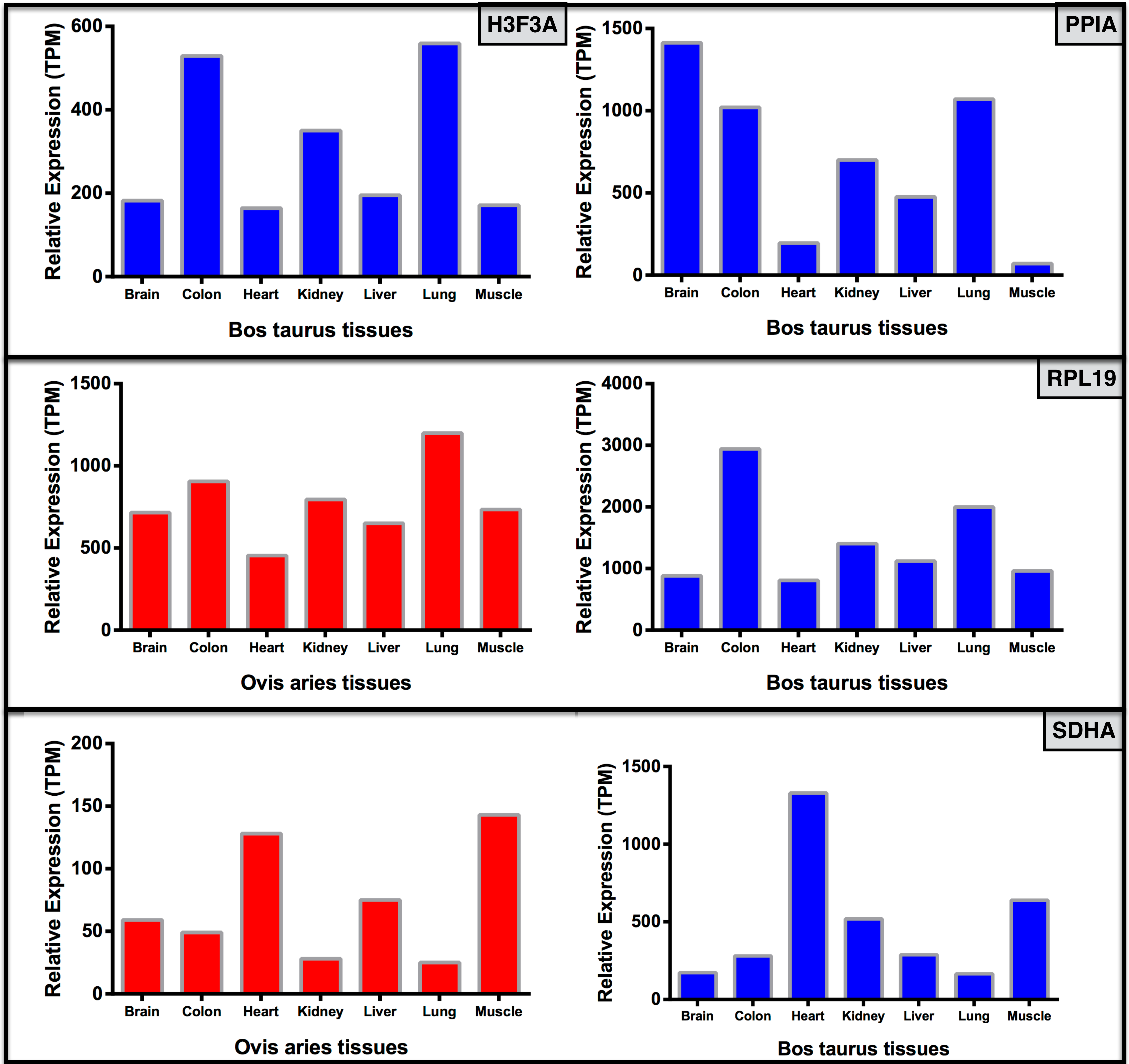

H3F3A
PPIA
RPL19
SDHA
Relative Expression (TPM)
Brain
Colon
Heart
Kidney
Liver
Lung
Muscle
Bos taurus tissues
Ovis aries tissues

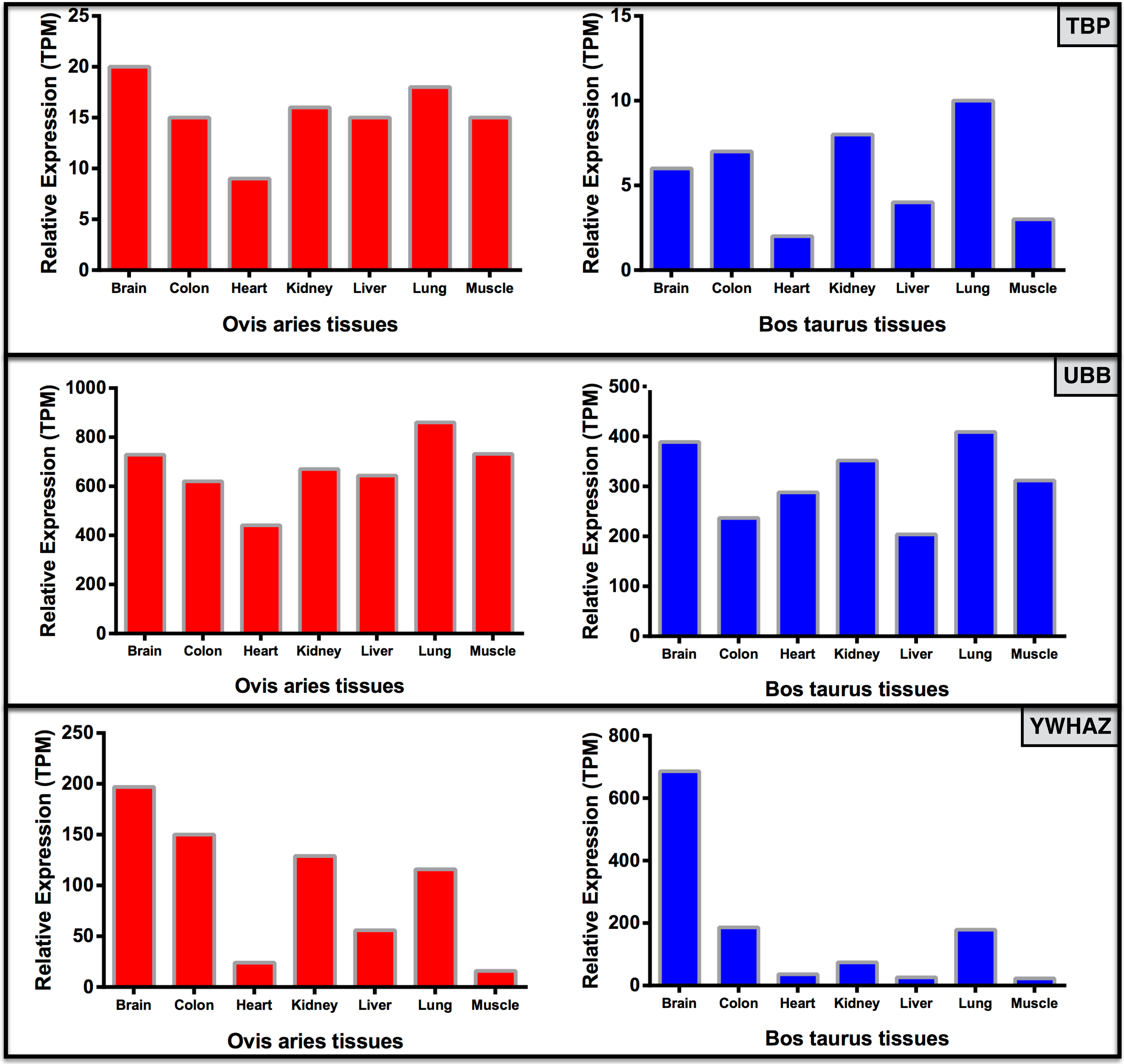
TBP
UBB
YWHAZ
Relative Expression (TPM)
Brain
Colon
Heart
Kidney
Liver
Lung
Muscle
Ovis aries tissues
Bos taurus tissues
0
5
10
15
20
25
100
200
300
400
500
600
800
1000
50
150
250
